# *Plasmodium* infection inhibits tumor angiogenesis through effects on tumor-associated macrophages in a murine implanted hepatoma model

**DOI:** 10.1186/s12964-020-00570-5

**Published:** 2020-09-24

**Authors:** Benfan Wang, Qinyan Li, Jinyan Wang, Siting Zhao, Bayaer Nashun, Li Qin, Xiaoping Chen

**Affiliations:** 1 State Key Laboratory of Respiratory Disease, Center of Infection and Immunity, Guangzhou Institutes of Biomedicine and Health, Chinese Academy of Sciences, Guangzhou, 510530 China; 2 School of Life Science, University of Science and Technology of China, Hefei, 230026 China; 3 CAS-Lamvac Biotech Co., Ltd, Guangzhou, 510530 China

**Keywords:** *Plasmodium* infection, Hepatocellular carcinoma, Growth inhibition, Tumor angiogenesis, Tumor-associated macrophages, Matrix metalloprotease, Hemozoin

## Abstract

**Background:**

Hepatocellular carcinoma (HCC) is one of the leading causes of cancer-related death in China. The lack of an effective treatment for this disease results in a high recurrence rate in patients who undergo radical tumor resection, and the 5-year survival rate of these patients remains low. Our previous studies demonstrated that *Plasmodium* infection provides a potent antitumor effect by inducing innate and adaptive immunity in a murine Lewis lung carcinoma (LLC) model.

**Methods:**

This study aimed to investigate the inhibitory effect of *Plasmodium* infection on hepatocellular carcinoma in mice, and various techniques for gene expression analysis were used to identify possible signal regulation mechanisms.

**Results:**

We found that *Plasmodium* infection efficiently inhibited tumor progression and prolonged survival in tumor-bearing mice, which served as a murine implanted hepatoma model. The inhibition of tumor progression by *Plasmodium* infection was related to suppression of tumor angiogenesis within the tumor tissue and decreased infiltration of tumor-associated macrophages (TAMs). Further study demonstrated that matrix metalloprotease 9 (MMP-9) produced by TAMs contributed to tumor angiogenesis in the tumor tissue and that the parasite-induced reduction in MMP-9 expression in TAMs resulted in the suppression of tumor angiogenesis. A mechanistic study revealed that the *Plasmodium*-derived hemozoin (HZ) that accumulated in TAMs inhibited IGF-1 signaling through the PI3-K and MAPK signaling pathways and thereby decreased the expression of MMP-9 in TAMs.

**Conclusions:**

Our study suggests that this novel approach of inhibiting tumor angiogenesis by *Plasmodium* infection is of high importance for the development of new therapies for cancer patients.

Video abstract

## Introduction

*Plasmodium* infection, which causes malaria in humans and animals, modulates the host’s immunity by inducing the secretion of cytokines, by activating some immune cell populations, and particularly by altering the function of macrophages [1, 2]. Therefore, Greentree proposed the notion of macrophage activation-based therapeutic malaria for the treatment of cancer in 1981 [3]. Angsubhakorn and colleagues found that *Plasmodium* infection reduced hepatic carcinogenesis induced by dietary aflatoxin B1 [4]. Our previous study demonstrated that *Plasmodium* infection exerts a potent antitumor effect through the induction of innate and adaptive antitumor immune responses in a murine Lewis lung carcinoma (LLC) model [5].

Angiogenesis plays a central role in the invasion, growth and metastasis of solid tumors through the formation of new blood vessels from preexisting vessels [6]. The blockage of angiogenesis significantly inhibits tumor growth [7]. Our previous study demonstrated that *Plasmodium* infection inhibits tumor angiogenesis by releasing plasma exosomes that contain endogenous functional microRNAs in a murine LLC model [8]. These effects were observed in rodents infected with malaria parasites, and the *Plasmodium* species that infect mice are different from those that infect humans. The precise mechanisms underlying the inhibition of tumor angiogenesis by *Plasmodium* infection remain unclear.

Based on a series of pilot experiments, we hypothesized that *Plasmodium* infection in tumor-bearing mice might regulate tumor angiogenesis by modulating the function of macrophages. Macrophages comprise the major proportion of host-derived immune cells associated with most solid tumors and are key regulators of tumor angiogenesis [6, 9]. In the tumor microenvironment, tumor-associated macrophages (TAMs) are polarized into the alternatively activated phenotype (M2 type) and promote tumor angiogenesis through the production of several proangiogenic factors, such as matrix metalloproteinases (MMPs) [6, 9, 10]. MMP-2 and MMP-9 are well documented to play crucial roles in the process of angiogenesis, mainly through degradation of the extracellular matrix (ECM) [8, 11, 12]. A recent study showed that increased type 1 insulin-like growth factor (IGF-1) signaling is associated with upregulation of MMP-2 and MMP-9 expression [13–15]. Both the Ras/Raf/ERK pathway and the PI 3-kinase/Akt signaling pathway are crucial pathways involved in the regulation of IGF-1 functions [16–18].

Hemozoin (HZ, a malarial pigment) is a polymer of heme produced by *Plasmodium* parasites during hemoglobin degradation inside infected red blood cells (iRBCs) [19]. This pigment is released when an iRBC bursts and is rapidly engulfed by phagocytes [20]. HZ can naturally be engulfed by TAMs. Although in vitro studies have revealed that HZ modulates the production of inflammatory factors by murine macrophages and human monocytes [21], there is a paucity of data on the role of the modulatory effect of HZ engulfed by TAMs on the regulation of tumor development.

In the present study, we demonstrated that *Plasmodium* infection effectively inhibited tumor progression and prolonged survival in tumor-bearing mice. The inhibition of tumor progression by *Plasmodium* infection was associated with decreased tumor angiogenesis. We further demonstrated that TAMs that produced MMP-9 contributed to tumor angiogenesis in tumor tissue and that the reduction in MMP-9 expression in TAMs mediated by infection resulted in the suppression of tumor angiogenesis. A mechanistic study revealed that *Plasmodium*-derived HZ that accumulated in TAMs inhibited IGF-1 expression through the PI3-K and MAPK signaling pathways, which led to decreased expression of MMP-9 in TAMs. Our study suggests a novel method for inhibiting tumor angiogenesis through *Plasmodium* infection.

## Methods

### Mice, cells and parasites

Female C57BL/6 mice (aged 6–8 weeks) were purchased from Beijing Vital River Experimental Animals Co., Ltd. The murine Hepa1–6 hepatoma cell line was obtained from the First Affiliated Hospital of Sun Yat-Sen University and cultured in Dulbecco’s modified Eagle’s medium (DMEM) (GIBCO) supplemented with 10% fetal bovine serum (FBS), 1% penicillin and streptomycin. The murine H22 hepatoma cell line was purchased from the China Center for Type Culture Collection and maintained in RPMI 1640 medium supplemented with 10% FBS, 1% penicillin and streptomycin. The murine RAW264.7 macrophage cell line was kindly provided by Dr. Chiwei Huang and cultured in DMEM supplemented with 10% FBS, 1% penicillin and streptomycin. *Plasmodium yoelii 17 XNL* (*P.y*), a *Plasmodium* parasite strain that is not lethal to C57BL/6 mice, was kindly provided by BEI Resources (formerly the Malaria Research and Reference Reagent Resource Center, MR4) and was recovered in 7-week-old female C57BL/6 mice. The antibodies (Table S1) and gene specific primers (Table S2) in this paper can be found in supplementary material.

### Design of animal experiments

To evaluate the effects of *Plasmodium* infection on tumors in mice, mice were subcutaneously inoculated with 2 × 10^6^ Hepa1–6 or H22 cells in 100 μl of serum-free RPMI 1640 and simultaneously intraperitoneally (i.p.) inoculated with 5 × 10^5^ parasitized erythrocytes or uninfected RBCs as a control in 200 μl of saline. The palpable spherical tumor mass emerged 4–6 days after tumor cell inoculation. The tumor size was measured every 3 or 4 days using a caliper and calculated using the formula 0.52 × a × b^2^ (a: long diameter of the tumor and b: short diameter of the tumor). Blood samples were collected from the tail vein of the mice every 2 days for 30 days, and parasitemia was determined by analyzing a thin Giemsa-stained blood film. The mice were observed until death or until their tumor size reached 2000 mm^3^. In some experiments, the mice were sacrificed on day 8 or 17 after parasite inoculation for biopsy. The tumor tissue was used for further analysis. For assaying the blockage of parasite infection, *Plasmodium* parasites were killed with a dose of 10 mg of chloroquine per kilogram of mouse body weight on day 8 after infection, and the mice were then euthanized on day 17 for further analysis.

To establish the orthotopic tumor model, 50 μl of 1 × 10^6^ Hepa1–6 cells were injected into the left lobe of the liver of anesthetized mice. After recovery, the mice were i.p. inoculated with 5 × 10^5^ parasitized erythrocytes or uninfected RBCs. On day 17 after parasite inoculation, the mice were sacrificed for biopsy. The liver and spleen were removed and photographed, and the liver sections were stained with H&E.

### Alginate-encapsulated tumor cell assay

An in vivo alginate-encapsulated tumor cell assay was performed as previously described with slight modifications [22]. Briefly, 1.5% (W/V) sodium alginate solution was prepared in sterile saline. Hepal-6 or H22 hepatoma cells were harvested once 80% confluence was reached in cell culture. After centrifugation, the cells were resuspended in the sodium alginate solution to a concentration of 2 × 10^7^/ml. The alginate solution was extruded through a point-cut 200-μl tip to produce droplets (20 μl per droplet). The addition of droplets into a swirling solution of 250 mmol/l calcium chloride at 37 °C resulted in the formation of alginate beads containing tumor cells in the solution. After incubation in the CaCl_2_ bath for an additional 30 min, the beads were washed twice with saline, centrifuged and resuspended in 10% RPMI1640 at 37 °C.

Four alginate beads (1 × 10^5^ Hepal-6 or H22 cells per bead) were subcutaneously implanted into an incision made on the dorsal side of the mice (aged 6–8 weeks) under anesthetic conditions. The mice were randomly divided into two groups (*n* = 10 per group). One group was i.p. inoculated with 5 × 10^5^ parasitized erythrocytes, and the other group, which served as the control, was i.p. inoculated with the same number of uninfected RBCs. After 14 days, the mice were i.v. injected with 0.1 ml of 1% FITC-dextran (Sigma-Aldrich) solution (100 mg/kg) through the lateral tail vein. After 20 min, the beads were rapidly removed and photographed. After dissection of the capsular implant, the beads were transferred to tubes containing 2 ml of saline. The tubes were mixed by vortexing for 30 s and centrifuged (5 min, 1000×g). After dilution (1:1), the fluorescence of the supernatant was measured. The uptake of FITC-dextran was assessed using a standard FITC-dextran curve. After removal of the implants from the mice, the following procedures were performed in the dark.

### TAM isolation

Single-cell suspensions were prepared from tumor tissue as described previously with some modifications [23]. Briefly, tumor-bearing mice were sacrificed 17 days after parasite inoculation. The solid tumors were dissected, chopped into small pieces using scissors and incubated with a mixture of enzymes dissolved in RPMI 1640 (400 U/ml collagenase type IV, 0.05 mg/ml collagenase type I, 0.025 mg/ml hyaluronidase, all from Sigma-Aldrich; 0.01 mg/ml DNase I and 0.2 unit/ml soybean trypsin inhibitor, both from Boehringer Mannheim) for 30 min at 37 °C. The cells were harvested by centrifugation. The RBCs were lysed using ammonium chloride lysing buffer. The suspension was filtered through a 70-μm BD Falcon cell strainer (BD Biosciences) to generate a single-cell suspension. The single-cell suspension was centrifuged, washed twice with phosphate-buffered saline (PBS) containing 1% FBS and resuspended in PBS containing 1% FBS and 2 mM EDTA. The cell suspension was layered onto Ficoll-Paque™ PLUS (GE Healthcare) and centrifuged at 450 g for 30 min without breakage. Mononuclear cells were obtained by removing the middle white layer, washed twice with phosphate-buffered saline (PBS) containing 1% FBS and resuspended in PBS containing 1% FBS. F4/80+ cells were isolated from the cell suspension by flow cytometry (BD FACSAria). Briefly, a total of 1 × 10^7^ cells were incubated with 10 μg of FITC- or APC-conjugated anti-F4/80 monoclonal antibody (mAb) for 30 min on ice and then washed with cold buffer to remove any unbound antibody. The F4/80^+^ cell populations were sorted by flow cytometry (BD FACSAria). The purity of the cell populations was between 85 and 90%.

### Induction of TAMs in vitro

Murine nonhypoxic TAMs were induced in vitro by incubation with soluble factors released from the murine hepatoma cell line Hepa1–6 as described previously with some modifications [24].

Tumor cell-free supernatant (TSN) was obtained by culturing 4 × 10^6^ Hepa1–6 cells in 24-well flat-bottom tissue culture plates for 72 h and stored in aliquots at − 80 °C. For in vitro induction of the TAM phenotype, 5 × 10^5^ RAW 264.7 cells were seeded in a six-well plate, and TSN (diluted 1:1 with fresh RPMI 1640 medium supplemented with 1% FBS) was added. After 48 h, the supernatant was removed, and the cells were washed twice with PBS. Total RNA was isolated using the TRIzol reagent according to the manufacturer’s instructions (Invitrogen). The expression levels of alternatively activated genes (*ym1, mgl1/2,* and *arginase 1*) and classically activated genes (*il-12* and *inos*) were analyzed by real-time qRT-PCR.

### Microarray experiment

Hepa1–6 cell-implanted mice were sacrificed on day 17 after *Plasmodium* infection. TAM isolation was performed as described in a previous section. The TAMs were sorted by FACS using FITC-conjugated anti-F4/80 mAb. Gene expression was compared using Roche NimbleGen mouse gene chips (CapitalBio, China). Genes that showed an expression fold-change ≥2 with a q-value ≤0.05 in triplicate arrays were considered to be significantly induced in response to treatment. These differentially expressed genes were annotated using the Molecule Annotation System (CapitalBio, China).

### Immunohistochemistry

The preparation and immunostaining of histological specimens were performed as described previously [25].

Paraffin-embedded tissue sections were deparaffinized in xylene and rehydrated through a graded ethanol series. High-pressure antigen retrieval was performed in 10 mM EDTA buffer (pH 8.0) before the sections were incubated with a primary antibody against F4/80, a rat mAb against mouse macrophages (clone No. RM0029-11H3), an anti-CD31 antibody and an anti-MMP-9 antibody. Bright field images were captured and analyzed.

The number of TAMs, density of CD31-positive microvessels and expression of MMP-9 in TAMs were assessed under 400× magnification (0.17-mm^2^ field) in eight random fields, and the results are expressed as the mean numbers per field ± SDs (eight fields per tumor, four tumors per group).

### RNA isolation and real-time RT-PCR

Total RNA was isolated using TRIzol (Invitrogen) according to the manufacturer’s instructions. Real-time RT-PCR for the *ym1*, *arg-1*, *mgl1*, *mgl2*, *fizz1*, *inos*, *mmp-2*, *mmp-9*, *leyve1*, *vegf*, *igf1,* and *β-actin* genes was performed with an MJ chromo 4 real-time PCR machine (Bio-Rad) using the OneStep SYBR® PrimeScript™ RT-PCR Kit (Takara) according to the manufacturer’s instructions. The *β-actin* gene was used as a reference gene.

### Western blot analysis

The preparation of the samples from tissue or cells, gel electrophoresis, and transfer to polyvinylidene fluoride membranes (Millipore) were performed as described previously [23]. The membranes were incubated with primary antibodies against MMP-2/9, VEGF, IGF-1, AKT, phospho-AKT, p44/42 MAPK (Erk1/2), and phospho-p44/42 MAPK (Erk1/2) overnight at 4 °C and with secondary antibodies for 1 h at room temperature. Immobilon Western HRP Chemiluminescent Substrate (Cell Signaling Technology) was used to detect the specific signals. The band intensity was visualized.

### Preparation of HZ

Natural HZ was obtained as described previously with some modifications [26]. Spleen and liver tissues of *P.y*-infected C57BL/6 mice were chopped into small pieces using scissors and lysed with 1% saponin for 2 h, and the resulting suspension was centrifuged (14,000 g, 10 min, 10 °C). The precipitate was washed three times with PBS by centrifugation. The precipitate was resuspended in 2% SDS and shaken for 1 h. The suspension was sonicated (3 s/time, 60 times) and centrifuged (25,000 g, 30 min at 10 °C). The precipitate was the crude extract of HZ, and this crude extract was further processed through the following steps. The precipitate was washed eight times with 2% SDS by centrifugation (25,000 g, 30 min, 10 °C), resuspended in 10 ml of 10 mM Tris-HCl (pH 8.0) containing 0.5% SDS, supplemented with proteinase K (2 mg/ml), DNase I (30 u/ml), and RNase A (10 mg/ml) and shaken overnight at 37 °C. The suspension was centrifuged (25,000 g, 30 min, 10 °C) and washed five times with 2% SDS by centrifugation (25,000 g, 30 min, 10 °C). The precipitate was resuspended with 6 M urea and shaken for 30 min at room temperature. The suspension was centrifuged (25,000 g, 30 min, 10 °C) with 2% SDS, and the precipitate was washed five times with 2% SDS by centrifugation (25,000 g, 30 min, 10 °C). After centrifugation, endotoxin contained by the extract was removed by addition of an endotoxin-binding reagent (Thermo Fisher Scientific). The extract was then washed with ultrapure H_2_O by centrifugation until complete removal of SDS was achieved. The pigment was lyophilized, dried, resuspended in endotoxin-free PBS at a final concentration of 10 mg/ml and maintained at − 20 °C. The endotoxin level of the isolated HZ was assessed using a *Limulus amebocyte* lysate kit (Sigma-Aldrich). The endotoxin level was less than 0.015 EU.

### Assessment of IGF-1 derived from HZ-loaded TAMs

To assess the effect of HZ on the secretion of IGF-1, induced TAMs were treated with HZ (100 μg/ml) or left untreated for 48 h. The IGF-1 mRNA and protein levels were evaluated by real-time PCR and immunoblot analysis, respectively. In some experiments, the cells were treated with different concentrations of HZ (10 to 200 μg/ml) for different durations (3 to 48 h).

### Preparation of dichloromethylene diphosphonate (Cl_2_MDP) liposomes and macrophage “*in vivo* knockout” assay

These experiments were performed by following previously described procedures [10]. A mixture of 8 mg of cholesterol and 86 mg of phosphatidylcholine was prepared in chloroform. The thin film was dissolved in 10 ml of 0.7 M Cl_2_MDP solution, incubated for 2 h at room temperature under the protection of argon, sonicated for 3 min and incubated for 2 h at room temperature. Free drugs were removed by centrifugation, and the final pellet was resuspended in PBS.

Naïve mice (aged 6–8 weeks) were i.p. injected with 300 μl of C1_2_MDP-liposomes and inoculated with tumor cells 2 days later. After tumor inoculation, the mice were treated with Cl_2_MDP-liposomes via both the intraperitoneal (300 μl) and intratumoral (50 μl) routes on days 4, 8, 13, and 18. PBS-liposomes and saline were used as controls. The tumor size was measured every 4 days and calculated. After 16 days, tumor-bearing mice were sacrificed for further analysis.

### Statistical analysis

The statistical significance of the differences between values was determined by Student’s t test using GraphPad Prism 6.01 software. The correlation between two factors was evaluated by Pearson’s correlation analysis. Kaplan-Meier survival curves were plotted. The data are expressed as the means ± SDs, and *P* values < 0.05 were considered significant.

## Results

### *Plasmodium* infection inhibited the growth of liver Cancer cells and prolonged the survival of tumor-bearing mice

**Fig. 1:**
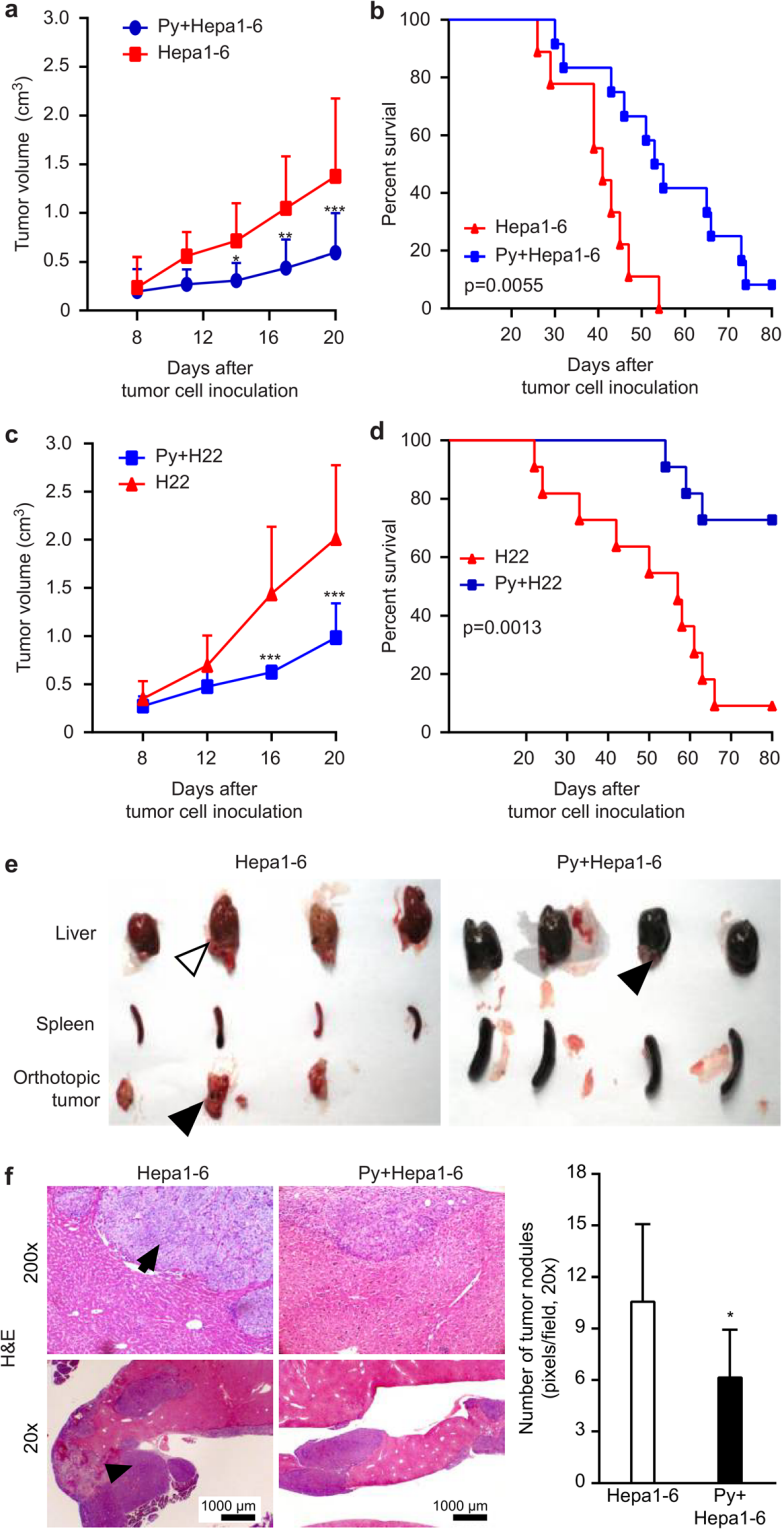
*Plasmodium* infection inhibited tumor progression and prolonged survival in tumor-bearing mice. **a**-**d** C57BL/6 mice were simultaneously administered a s.c. injection of Hepa1–6 (**a**, **b**) or H22 (**c**, **d**) cells and an i.p. injection of *Plasmodium* parasites (*n* = 12). The tumor sizes and survival of the mice were monitored. The data are representative of three independent experiments. The columns present the means ± SDs. The statistical analysis was performed using GraphPad Prism software 6.0 (two-way analysis of variance (ANOVA)). **, *p* < 0.01; ***, *p* < 0.001. **e**-**f** Hepa1–6 cells were injected into the livers of mice, and parasites were inoculated via the i.p. route (*n* = 10). Seventeen days later, the liver, spleen, and orthotopic tumor (black arrow) were removed and photographed (**e**). The nodules (black arrow) in the liver were observed by H&E staining and imaged (**f**, left panel). Original magnification: 400×. The number of nodules was counted under the same original magnification (200×) for quantitative analysis (**f**, right panel)

To observe the effect of *Plasmodium* infection on tumor growth, aged 6–8 week mice were simultaneously administered a subcutaneous (s.c.) injection of Hepa1–6 or H22 cells and an intraperitoneal injection of the malarial parasite. The tumor growth and survival rates of the tumor-bearing mice were evaluated. As shown in Fig. 1a, *Plasmodium* infection significantly inhibited tumor growth in the Hepa1–6 cell-implanted hepatoma model. The survival rate of the tumor-bearing mice in the infected group was significantly higher than that of the tumor-bearing mice in the uninfected control group (Fig. 1b). Considering the potential side effects of *Plasmodium* infection, parasitemia was monitored by thin Giemsa-stained blood film microscopy. The parasite density appeared to peak on days 16 to 24 and then decreased to a low level until it disappeared on day 32 (Fig. S1).

The inhibitory effect of *Plasmodium* infection on tumor growth was also observed in the H22 cell-implanted hepatoma model (Fig. 1c). The survival rate of the tumor-bearing mice in the infected group was also significantly higher than that of the tumor-bearing mice in the control group (Fig. 1d).

To further validate the inhibitory effect of *Plasmodium* infection on tumor growth, we performed a similar experiment using an orthotopically implanted tumor model. Gross observation of the removed tumors (Fig. 1e) and microscopic observation of hematoxylin and eosin (H&E)-stained tumor tissue (Fig. 1f, left panel) yielded a similar result. The infected group exhibited significantly reduced tumor nodule formation in the liver compared with the control group (Fig. 1f, right panel).

### *Plasmodium* infection suppressed tumor angiogenesis

**Fig. 2:**
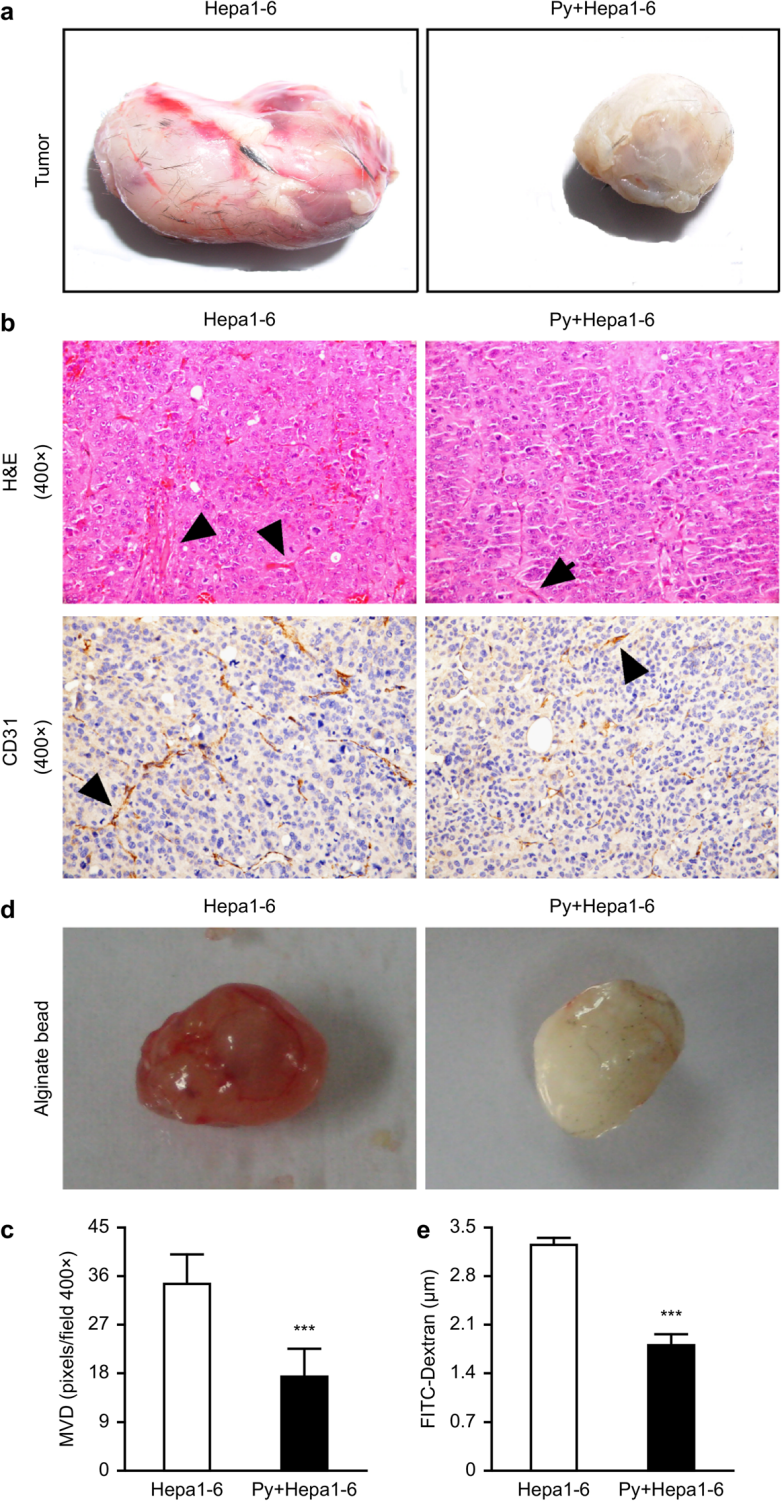
*Plasmodium* infection suppressed neovascularization in Hepa1–6-implanted tumor tissue. **a**-**c** The vascularization of tumor tissue from tumor-bearing mice on day 17 after *Plasmodium* infection was imaged (*n* = 4) (**a**). H&E staining showed neovascularization of the tumor in tumor-bearing mice on day 17 after *Plasmodium* infection (*n* = 4) (**b**, upper panel). Representative images (**b**, bottom panel) and quantitative analysis (**c**) of the microvessel density (MVD) by immunohistochemical staining with antibodies reactive to CD31 were used to characterize the degree of neovascularization in tumor-bearing mice on day 17 after *Plasmodium* infection. Original magnification: 400×. ***, *p* < 0.001. **d**-**e** The alginate-encapsulated tumor cell assay was performed as described in the “Materials and methods” section (*n* = 10). Representative images of the alginate beads are shown (**d**). The FITC-dextran uptake by the beads was quantified (**e**). The data are expressed as the means ± SDs. ***, *p* < 0.001

It is worth noting that tumor angiogenesis was significantly inhibited and tumor growth was inhibited in the *Plasmodium*-infected tumor-bearing mice (Fig. 2a). Consistent with this result, H&E staining and CD31 immunohistochemical staining of the tumors from tumor-bearing mice on day 17 after parasite infection showed that tumor angiogenesis was significantly decreased in the infected mice compared with the control mice (Fig. 2b and c).

To further confirm whether *Plasmodium* infection might lead to the inhibition of angiogenesis in tumor-bearing mice, an alginate-encapsulated tumor cell assay was performed. As shown in Fig. 2d, angiogenesis was markedly inhibited in the hepatoma tissue of Hepa1–6 cell-implanted mice infected with the parasite. The uptake of FITC-dextran uptake was significantly lower in the tumor-bearing mice infected with the parasite than in the control mice (Fig. 2e). Together, these results showed that *Plasmodium* infection induced an important reduction in tumor angiogenesis in tumor-bearing mice, which led to the inhibition of tumor growth.

### Suppression of TAMs mediated by *Plasmodium* infection contributed to the inhibition of tumor angiogenesis

**Fig. 3:**
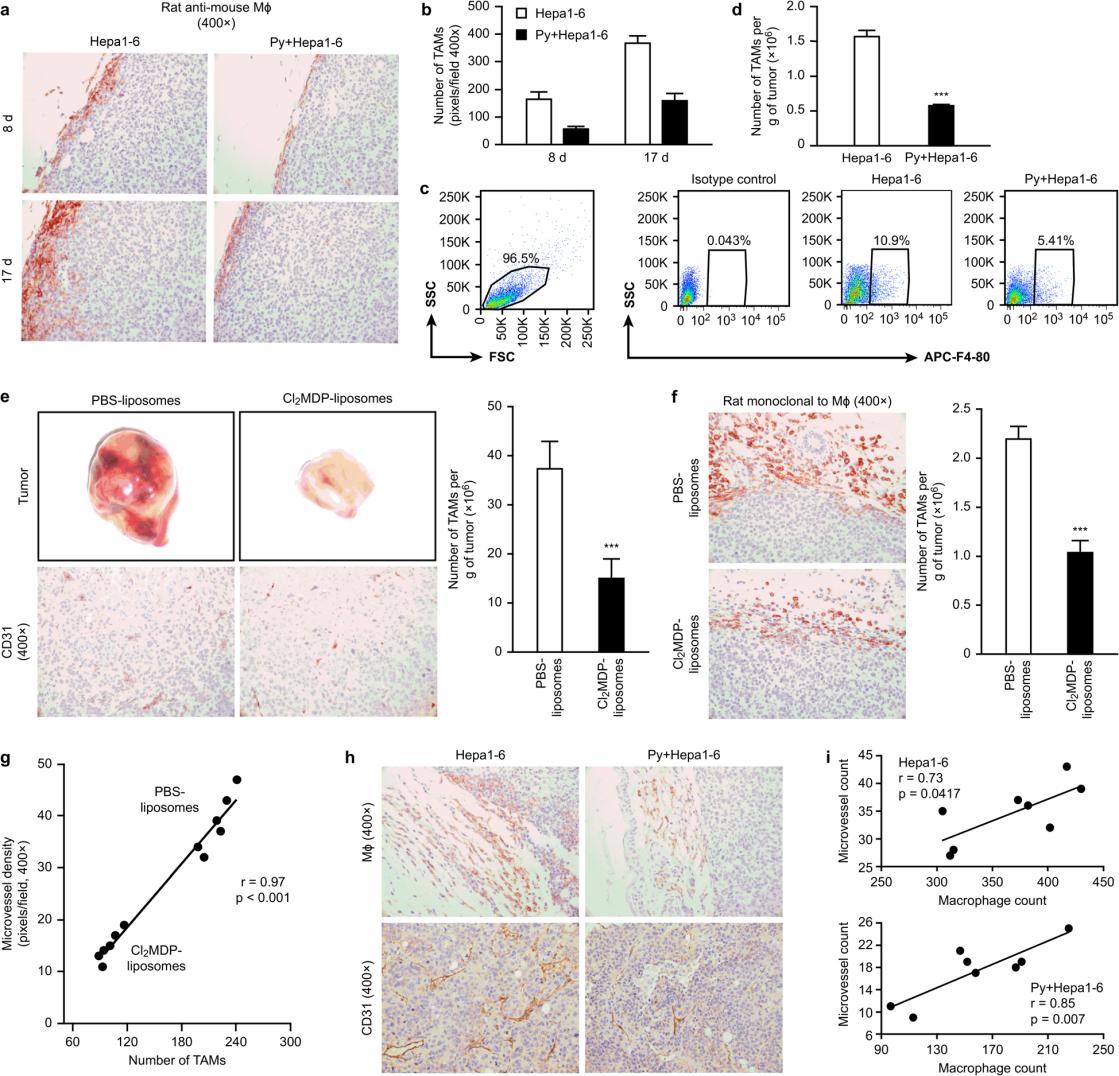
Quantification of TAM infiltration into tumor tissue. **a**-**b** Tumor histological specimens were prepared on days 8 and 17 after infection with *Plasmodium* parasites (*n* = 4) and stained immunohistochemically with a “rat mAb against mouse macrophages” (clone No. RM0029-11H3) (colored by AEC). Representative images are presented (**a**). The number of infiltrating TAMs was quantified by counting (**b**). ***, *p* < 0.001. **c**-**d** The infiltrating TAMs were sorted by FACS using a FITC-conjugated anti-F4/80 mAb. The proportion of TAMs was analyzed (**c**). The absolute number of TAMs was quantified per gram of tumor (*n* = 5) (**d**). All the data were analyzed using FlowJo software (7.6.1 version). The purity of the cell populations was between 85 and 90%. **e**-**f** Hepa1–6 tumor-bearing mice were treated with Cl_2_MDP liposomes at the scheduled time point. The tumor tissues were dissected after the mice were sacrificed, and tumor angiogenesis was examined visually and imaged (**e**, upper panel). PBS liposomes were used as a control. Neovascularization was examined by immunohistochemical staining with antibodies reactive to CD31, and representative images are shown (**e**, bottom panel). The infiltrating TAMs were stained with a rat mAb that recognizes mouse macrophages [RM0029-11H3] and imaged (**f**, left panel). Original magnification: 400×. Quantitative analyses of the MVD (**e** right panel) and TAMs (**f**, right panel) were performed. The data are presented as the mean numbers of MΦ/MVD per field ± SDs (10 fields per tumor sample, six tumors per group, 400× magnification, 0.17 mm^2^/field). ***, *p* < 0.001. **g** The correlation between TAM infiltration and MVD in tumor-bearing mice treated with Cl_2_MDP liposomes or PBS liposomes was analyzed by Pearson’s correlation analysis. **h**-**i** Histological tumor specimens were prepared on day 17 after infection with *Plasmodium* parasites (*n* = 4) and stained immunohistochemically with a “rat mAb against mouse macrophages” and an antibody reactive to CD31. Representative images are shown (**h**). Original magnification: 400×. The number of infiltrating TAMs and the MVD were quantified by counting. The data are presented the numbers per field ± SDs (10 fields per tumor sample, eight tumors per group, 400× magnification, 0.17 mm^2^/field). The correlation between TAM infiltration and the MVD was analyzed by Pearson’s correlation analyses (**i**)

TAMs are reportedly important promoters of tumor angiogenesis in the tumor microenvironment [6]. To determine the effect of TAMs on tumor angiogenesis in the *Plasmodium* infection model, we analyzed the infiltration of TAMs into the tumor tissue on days 8 and 17 after infection. Immunohistochemical staining showed that *Plasmodium* infection significantly decreased the number of TAMs on days 8 and 17 after infection compared with the number obtained with the control treatment (Fig. 3a and b; Fig. S2). Furthermore, the proportion of TAMs in the uninfected tumor-bearing controls was approximately 11%, whereas this proportion was markedly decreased to approximately 5% in the tumor-bearing mice on day 17 after parasite infection (Fig. 3c). Examination of the absolute number of TAMs revealed that parasite infection significantly reduced the number of infiltrating TAMs (Fig. 3d).

Our previous study demonstrated that the reduction of TAM infiltration induced by treatment with Cl_2_MDP liposomes led to an improvement in the tumor microenvironment and a significant inhibition of tumor growth [10]. To further confirm that a reduction in TAM infiltration might induce inhibition of tumor angiogenesis, we designed a TAM “*in vivo* knockdown” assay by treating tumor-bearing mice with Cl_2_MDP liposomes. The blood vessel density and CD31 expression were obviously reduced in the tumors treated with Cl_2_MDP liposomes compared with the phosphate-buffered saline (PBS) liposome-treated tumors (Fig. 3e). Consistent with this result, the number of MF+ macrophages in the tumors treated with Cl_2_MDP liposomes was also significantly decreased (Fig. 3f). Moreover, an analysis of the correlation between the expression of CD31 and that of MF molecules in the tumor microenvironment of the Cl_2_MDP liposome-treated tumor-bearing mice revealed that the extent of tumor angiogenesis was positively correlated with the number of infiltrating TAMs in the tumor (Fig. 3g). Interestingly, compared with the uninfected control group, the infected group exhibited reduced tumor angiogenesis and decreased TAM infiltration (Fig. 3h and i).

Taken together, our data indicate that TAMs contribute to tumor angiogenesis, which is inhibited by *Plasmodium* infection.

### *Plasmodium* infection reduced the expression of IGF-1 and MMP-9 in TAMs

**Fig. 4:**
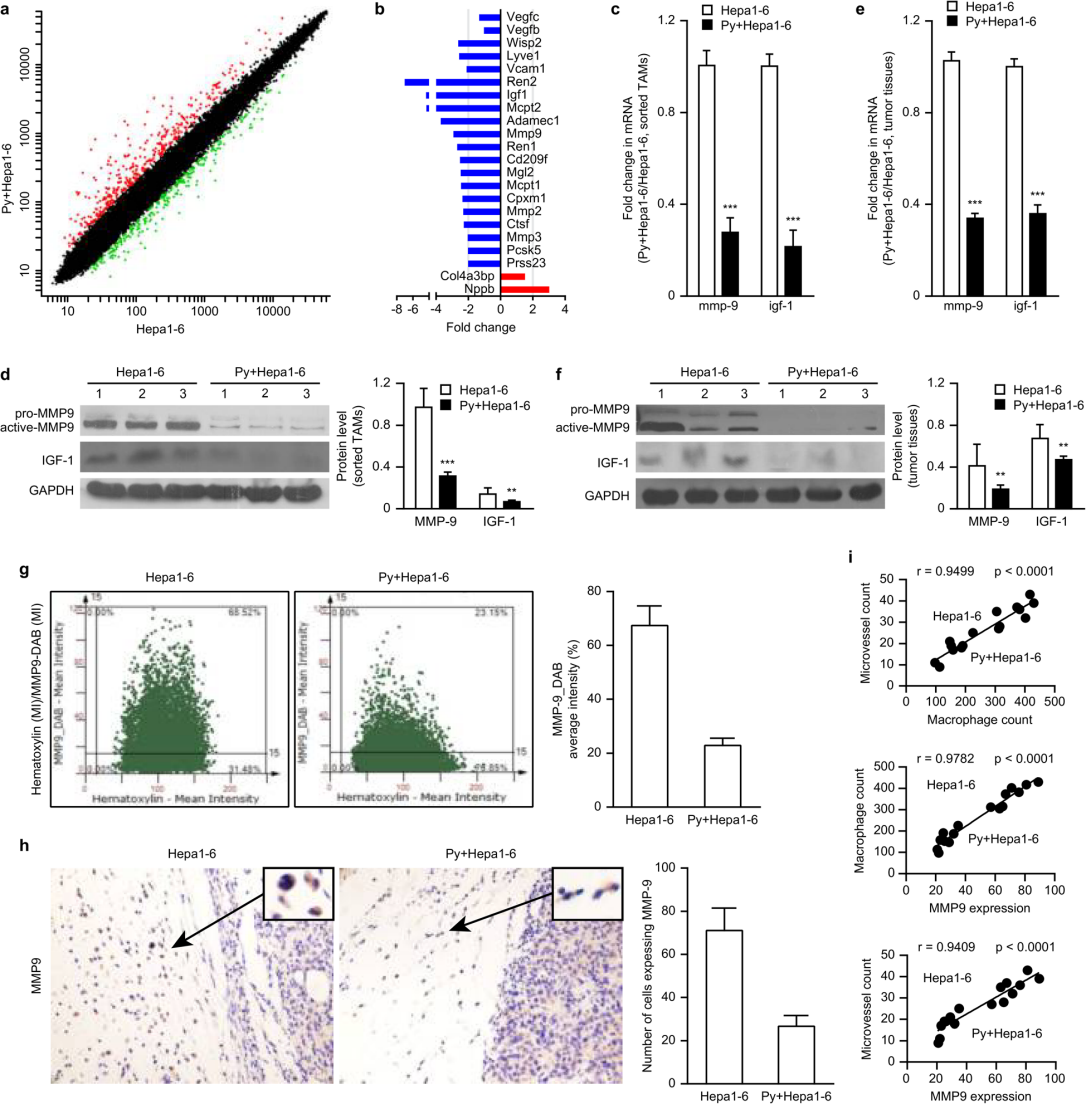
Attenuated MMP-9 expression in the infiltrating TAMs in tumor-bearing mice infected with *Plasmodium* parasites led to inhibition of tumor angiogenesis. **a**-**b** Tumor-bearing mice were sacrificed on day 17 after infection with *Plasmodium* parasites. TAMs were sorted, RNA was extracted, and the gene expression in TAMs was then detected through a gene expression (Roche NimbleGen) analysis. Genes with a ≥ 2-fold difference in expression and a q-value ≤0.05 were selected and mapped (**a**). A molecular annotation system was used to analyze the functions of these differentially expressed genes and the biological processes involved. Genes associated with tumor angiogenesis and that had a ≥ 2-fold difference in expression and a q-value ≤0.05 were selected for further analysis (**b**). **c**-**f** The relative MMP-9 and IGF-1 mRNA levels in the sorted TAMs (**c**) and tumor tissue (**e**) were quantified by qRT-PCR. A Western blotting analysis was performed to visualize the expression of MMP-9 and IGF-1 in the sorted TAMs (**d**, left panel) and tumor tissue (**f**, left panel). A quantitative analysis was performed using ImageJ software to quantify the endogenous MMP-9 and IGF-1 levels in the sorted TAMs (**d**, right panel) and tumor tissue (**f**, right panel). **g** A FACS-like tissue cytometer analysis system was used to analyze MMP-9 expression in the cells in the tumor margin by immunohistochemical staining with an antibody reactive to MMP-9 on day 17 after infection with *Plasmodium* parasites. The double-positive scatter plots represent the MMP-9-positive cells in scattergrams (left panel). The percentages represent the ratio of MMP-9-positive cells to total cells in the selected area. The mean intensity (MI) represents the average DAB intensity (right panel). **h** Representative images of MMP-9-positive cells identified by immunohistochemical staining with an antibody reactive to MMP-9 on day 17 after infection with *Plasmodium* parasites (original magnification: 400×). The insets show representative cells expressing MMP-9 (left panel) and the number of cells expressing MMP-9 (right panel). **i** A comprehensive analysis of the correlation among MMP-9 expression, MVD, and macrophage infiltration in the tumors of tumor-bearing mice on day 17 after *Plasmodium* infection compared with that in the tumors from uninfected tumor-bearing mice. *p* < 0.05 was considered significant

To explore the underlying mechanism through which *Plasmodium* infection inhibits tumor angiogenesis mediated by TAMs, we sorted the TAMs from the murine tumors and performed a gene expression analysis. The purity of the sorted TAMs was approximately 90% (Fig. S3a). As expected, the infected group exhibited markedly changed TAM gene expression compared with the control group (Fig. 4a, Fig. S3b). Importantly, significant differences in the gene expression profiles related to angiogenesis were found between the two groups. Lower expression of proangiogenic genes, such as *mmp-2/9* and *igf-1*, and higher expression of antiangiogenic genes, such as *Nppb*, was found in the infected group compared with the control group. Unexpectedly, the expression of the *vegf* gene in TAMs was slightly reduced in the infected group (Fig. 4d). A pathway analysis showed that *igf-1* was a key upstream regulator of *mmp-2/9*. These results were further confirmed by RT-PCR analyses and validated by Western blot assays. As expected, the RNA and protein levels of *mmp-9* in the TAMs from parasite-infected tumors were significantly lower than those in the TAMs from the control tumors. The levels of *igf-1* RNA and IGF-1 protein were also reduced approximately 4-fold in the infected mice (Fig. 4c and d), although the level of mmp-2 was only slightly reduced (Fig. S3c). These results suggested that the reduction in MMP-9 and IGF-1 expression in TAMs induced by *Plasmodium* infection might be responsible for the reduction in tumor angiogenesis.

To further investigate the concentrations of MMP-9 and IGF-1 within the tumor microenvironment, we also analyzed the tumor tissue 17 days after *Plasmodium* infection. Consistent with their expression in TAMs, MMP-9 and IGF-1 expression was also significantly decreased in the infected tumor tissue (Fig. 4e and f); however, the expression of VEGF was only slightly decreased in the infected tumors compared with the uninfected tumors (Fig. S3c). In addition, the results from a FACS-like tissue cytometry analysis confirmed that MMP-9 expression was significantly lower in the *Plasmodium*-infected group than in the control group (Fig. 4g, Fig. S4). Taken together, these results suggest that *Plasmodium* infection might reduce MMP-9 and IGF-1 expression in TAMs and tumor tissue and that this effect is responsible for the reduced angiogenesis within tumors.

### The number of TAMs and MMP-9 expression were positively correlated with tumor vascular density

An immunohistochemical analysis revealed that the cells secreting MMP-9 were mainly localized in the tumor margin, and most of these cells were macrophages (Fig. 4h). We subsequently performed correlation analyses using the expression levels of MMP-9, CD31 and MF molecules in the *Plasmodium*-infected model measured through immunohistochemical analysis. Pearson’s correlation analysis showed that MMP-9, the number of infiltrating TAMs, and the degree of angiogenesis achieved quantitatively similar positive correlations in the parasite-infected group (Fig. S5a) and the uninfected group (Fig. S5b). Furthermore, the combination of the two groups yielded similar quantitative results (Fig. 4i). Taken together, these results suggest that the reduction in MMP-9 expression in TAMs is significantly correlated with the inhibition of tumor angiogenesis in the *Plasmodium*-infected model.

### *Plasmodium* infection reduced IGF-1 expression by blocking IGF-1R signaling and the PI3-K and MAPK pathways

**Fig. 5:**
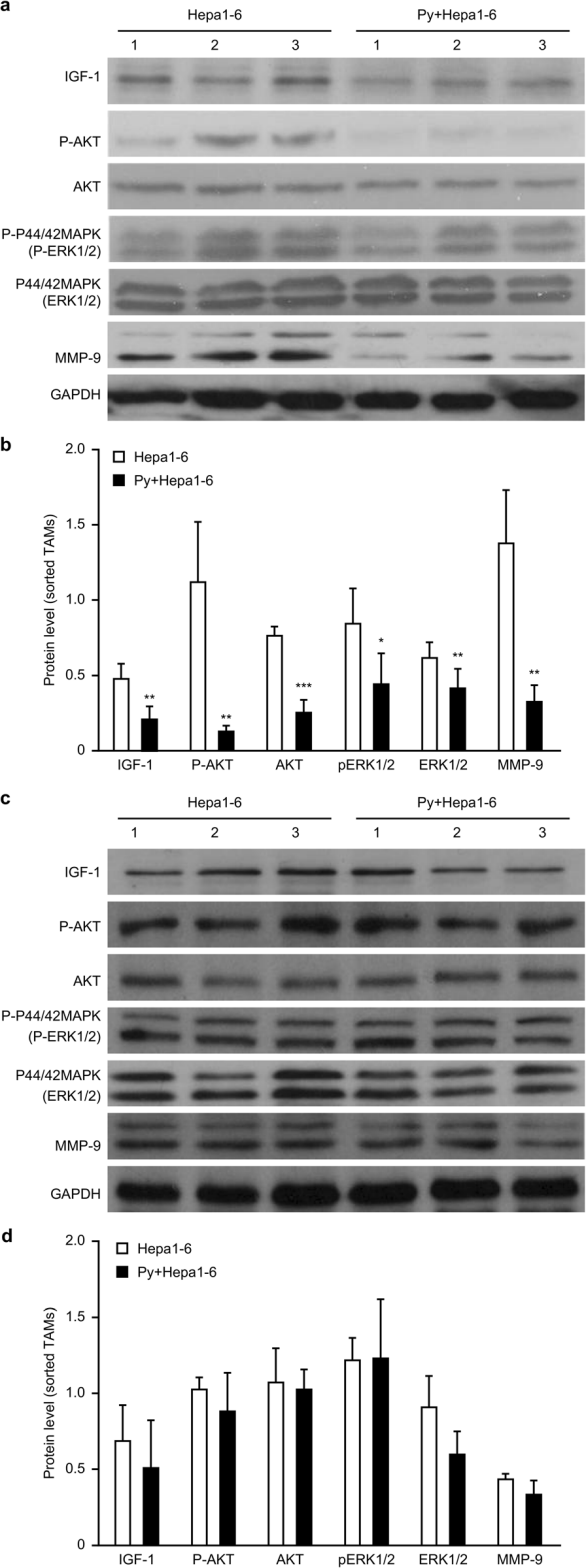
*Plasmodium* infection attenuated MMP-9 expression by blocking IGF-1R signaling. **a**-**b** The expression of IGF-1, MMP-9, phosphorylated Akt, total Akt, phosphorylated p42/44 MAPK and total MAPK in the sorted TAMs from tumor-bearing mice on day 17 after *Plasmodium* infection was detected by immunoblotting (**a**). A quantitative analysis was performed using ImageJ software to quantify the levels of these signaling proteins in the sorted TAMs (**b**). **c**-**d** The *Plasmodium* parasites were killed by chloroquine on day 8, and the AKT and MAPK signaling pathways were examined. A Western blotting assay was performed to visualize the restoration of the levels of these signaling proteins (**c**). A quantitative analysis of signaling proteins in the sorted TAMs from the tumor-bearing mice with *Plasmodium* infection blocked on day 8 was performed using ImageJ software (**d**)

IGF-1 reportedly promotes MMP-9 expression via the AKT and MEK/ERK pathways [13–16]. We subsequently conducted a mechanistic study to explore the role of IGF signaling in the expression of MMP-9 in *Plasmodium*-infected tumor-bearing mice and found a significant reduction in the p-AKT and phosphorylated p42/44 MAPK levels in the infected group compared with the uninfected group (Fig. 5a and b). The termination of parasite infection restored the expression of these signaling proteins (Fig. 5c and d), which implies that the PI3-K and MAPK pathways participate in IGF-1 signaling to induce MMP-9 expression.

### HZ, a *Plasmodium* metabolite, reduced the expression of IGF-1 in TAMs

**Fig. 6:**
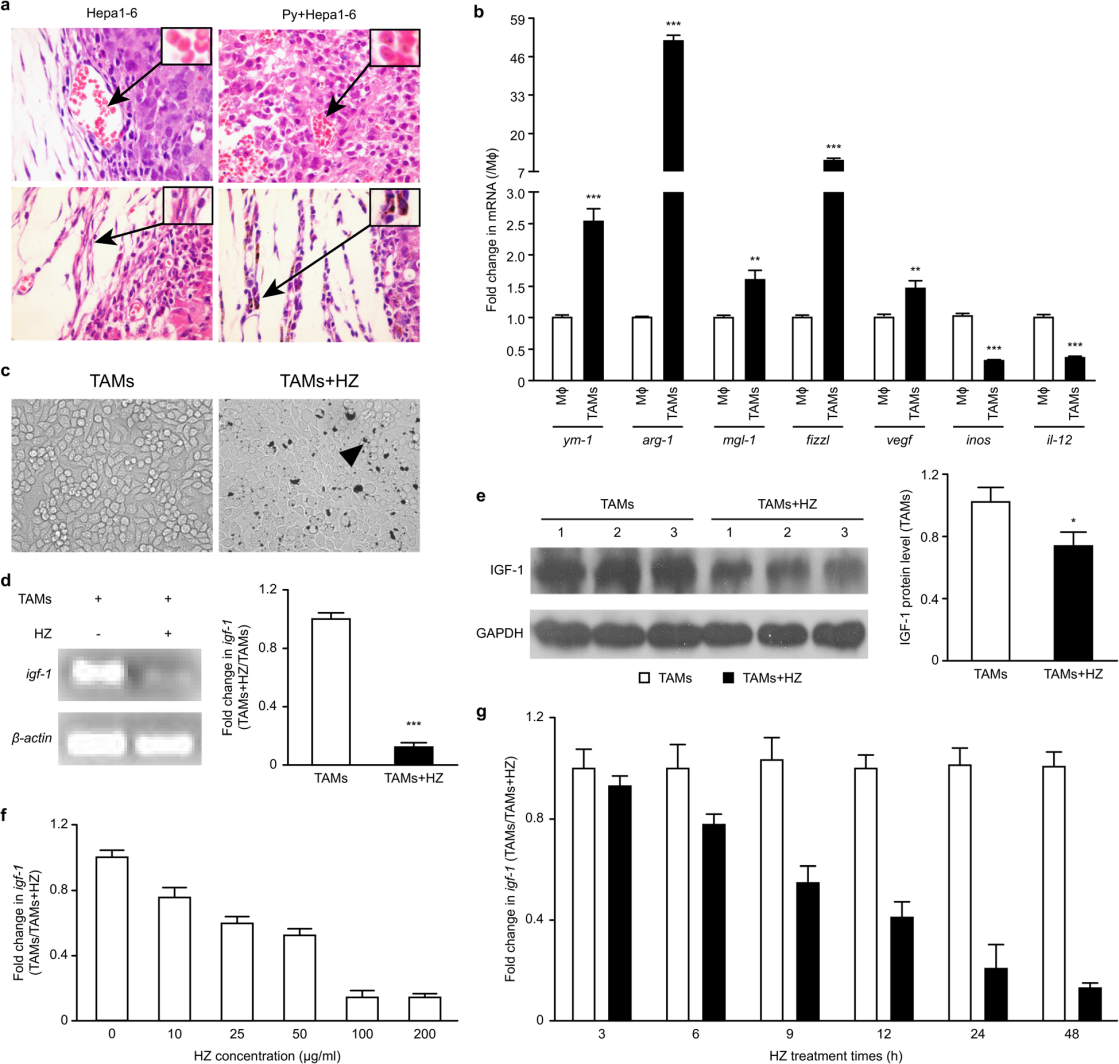
HZ modulated the expression of IGF-1 in TAMs polarized in vitro by coculture. **a** Tumor tissue sections were observed on day 17 after *Plasmodium* infection (*n* = 4). Parasite-infected red blood cells (iRBCs) could be found in the tumor tissue (upper panel). Representative photos are presented (1000× magnification). The insets show representative iRBCs, and the black arrows identify *Plasmodium* parasites in the iRBCs. HZ was found in TAMs from Hepa1–6 cell-implanted mice infected with *Plasmodium* parasites (n = 4) (bottom panel). The arrows indicate representative cells with HZ accumulation in their cytoplasm. Original magnification: 400×. RAW264.7 cells were plated in the absence or presence of TSN (1:2 dilution). **b**-**c** Genes associated with TAM phenotype characteristics were analyzed in the induced TAMs by real-time PCR (**b**). The phagocytosis of HZ by RAW264.7 cells in vitro was also observed (**c**). The arrows illustrate examples of cells with HZ accumulation in their cytoplasm. Original magnification: 400×. **d**-**e** The cells were treated with or without HZ (100 μg/ml) in the presence of TSN (1:2 dilution). The level of IGF-1 was analyzed 48 h after coculture by RT-PCR (d, left panel) and qRT-PCR (d, right panel). The expression of IGF-1 was detected by immunoblotting and quantified using ImageJ software (**e**). **f**-**g** In some experiments, the induced TAMs were treated with different doses of HZ, and 48 h later, the expression of IGF-1 mRNA was analyzed by qRT-PCR (**f**). In other experiments, the induced TAMs were exposed to HZ (100 μg/ml) for various time periods, and the level of IGF-1 was then analyzed (**g**). The results are presented as the means ± SDs from triplicate samples in three independent experiments

As expected, *Plasmodium*-infected red blood cells (iRBCs) were found within the tumors from the tumor-bearing mice (Fig. 6a, upper panel). Interestingly, we observed that HZ, a metabolite of the *Plasmodium* parasite, accumulated in TAMs (Fig. 6a, lower panel). We thus postulated that HZ can play roles in the modulation of IGF-1 expression in TAMs.

To confirm this hypothesis, we examined the expression of IGF-1 molecules in polarized M2-like macrophages stimulated with HZ. To generate M2-like macrophages, RAW264.7 cells were cocultured with TSN in vitro. These polarized cells showed enhanced expression of genes such as *ym1, fizzl, mgl1*, *arginase-1* (*arg-1*), and *vegf*, but the expression of *inos*, a gene that is correlated with classical activation, was downregulated (Fig. 6b). Furthermore, these polarized M2-like macrophages can phagocytose HZ in vitro (Fig. 6c), and the cell viability was not affected by the doses of HZ used.

Polarized M2-like macrophages were treated with HZ for 48 h, and the expression of the *igf-1* gene was examined. As shown in Fig. 6d and e, the expression of IGF-1 significantly decreased by the phagocytosis of HZ by M2-like macrophages. Interestingly, the expression of IGF-1 was reduced in a dose-dependent manner (Fig. 6f). Specifically, 100 μg/ml HZ induced a markedly stronger suppression of the expression of this gene in M2-like cells compared with a dose of 10 μg/ml. However, the treatment of M2-like cells with HZ (100 μg/ml) for the time periods indicated in Fig. 6g revealed that the change in IGF-1 expression was dependent on the treatment time. Together, these results show that HZ plays an important role in the reduction of IGF-1 expression in TAMs of *Plasmodium*-infected tumor-bearing mice.

## Discussion

The present study shows that *Plasmodium* infection efficiently inhibits hepatic tumor progression through inhibition of tumor angiogenesis mediated by TAMs. Mechanistic studies revealed that the *Plasmodium* product HZ, which is taken up by TAMs, downregulates MMP-9 expression by suppressing IGF-1 via the PI3-K and MAPK pathways.

After Anopheles mosquitoes bite humans or vertebrates, *Plasmodium* parasites move with the blood to the liver, mature and reproduce in liver cells, and significantly alter the physiological characteristics of the liver. Infection with the *Plasmodium berghei* (*P.b*) NK65 strain can cause excessive oxidative stress in the mouse liver, and *Plasmodium chabaudi* (*P.c*) AS infection causes liver inflammation in mice due to a cytokine burst and increases in serum liver enzymes [27, 28]. *P.y* parasites perturb the regulatory pathways in hepatocytes involved in cell survival, proliferation, and autophagy and suppress p53 activity in liver cells [29]. *Plasmodium falciparum* (*P.f*) parasites are able to accelerate the anaerobic hydrolysis of glucose in host cells to produce lactic acid, which might cause hypoglycemia and lactic acidosis [30]. These studies suggest that *Plasmodium* infection might induce changes in distinct physiological states at the liver stage.

Tumor cells usually exhibit increased metabolic activity compared with normal cells. To maintain the energy required for their high proliferation rate, tumor cells have a special metabolic phenotype, denoted the “Warburg Effect”, to survive in the tumor microenvironment, which involves a lack of nutrition and oxygen [31]. The metabolite lactate not only provides energy for tumor cell growth but also induces acidification of the tumor microenvironment and switches the functions of immune cells to tumor-promoting and angiogenesis-tolerant phenotypes [31, 32]. Studies have shown that lactate can induce the differentiation of macrophages into TAMs by modifying histone lysine acetylation [33]. In tumor-bearing mice, *Plasmodium* parasites can consume a high amount of glucose due to their rapid proliferation. As a result, tumor cells are starved due to a lack of energy sources, and tumor cell growth might thus be markedly restricted. In contrast, *Plasmodium* infection can lead to a cytokine burst, which could perturb the tumor microenvironment and reverse the dysfunction of immune cell infiltration in tumors. A study conducted by Pasquetto and colleagues revealed that *Plasmodium* infection can trigger the infiltration of a large number of NK cells, macrophages, and T cells in the liver. As a result, IFN-γ and IFN-α/β produced by these immune cells inhibit HBV gene expression and replication in the liver, which indicates that *Plasmodium* infection might exert a preventive effect on HBV-induced liver cancer [34].

It has long been recognized that parasite infection might serve to enhance immune surveillance mechanisms against some types of solid cancers [5, 8, 35]. Furthermore, our global epidemiological data analysis suggested an inverse correlation between malaria incidence and cancer mortality [35]. *Plasmodium* pathogen-associated molecular patterns (PAMPs), such as glycosylphosphatidylinositol (GPI) anchors, HZ and immunostimulatory nucleic acid motifs [36], can be recognized by host immune cell sensors called pattern recognition receptors (PRRs) [37, 38], and this recognition triggers systemic immune responses that counteract the immunosuppressive tumor microenvironment containing TGF-β, IL-10, regulatory T cells and myeloid-derived suppressive cells (MDSCs) and thereby contributes to the suppression of tumor development [39, 40]. Indeed, our previous study demonstrated that blood-stage malaria exerts antitumor effects by inducing a potent antitumor innate immune response that includes the secretion of IFN-γ and the activation of natural killer (NK) cells. Our murine lung cancer model studies also demonstrated that malaria infection induces adaptive antitumor immunity by increasing the proliferation of tumor-specific T cells, the cytolytic activity of CD8+ T cells and the infiltration of these cells into tumor tissue [5]. A study conducted by Deng and colleagues demonstrated that the inoculation of attenuated liver-stage *Plasmodium* induces an antitumor innate immune response that includes the secretion of tumor necrosis factor (TNF)-α, IL-6/12, and IFN-γ and an antitumor adaptive immunity characterized by increased CD8+ T cell cytolytic activity [41].

Macrophages or TAMs derived from circulating monocytes are often the most abundant immune cells in the tumor microenvironment and are key regulators of tumor progression [9, 10, 42]. Our previous study showed that TAM depletion mediated by clodronate liposomes inhibits hepatic tumor growth [9]. Indeed, the role of TAMs in promoting hepatoma growth has been confirmed by several clinical investigations [43, 44]. Therefore, in the present study, we focused on investigating the role of TAMs in tumor angiogenesis in tumor-bearing mice infected with *Plasmodium*.

Accumulating evidence indicates that TAMs release a panel of potent proangiogenic cytokines and growth factors, such as VEGF, TNFα, IL-8, basic fibroblast growth factor, thymidine phosphorylase, urokinase-type plasminogen activator, adrenomedullin, semaphoring 4D, and cyclooxygenase-2 [45–47]. VEGF is known to serve as a major proangiogenic cytokine released by TAMs that causes an angiogenic switch. Its levels show correlations with the density of TAMs and tumor angiogenesis in several types of human cancer [48]. However, our present study showed no significant decrease in the VEGF levels in TAMs in *Plasmodium*-infected tumor-bearing mice, which suggests the potential existence of other antiangiogenic mechanisms. Several studies have demonstrated that TAMs might produce some angiogenesis-related enzymes, including MMP-2 and MMP-9, which mediate ECM degradation and increase the vascular invasion of tumor cells [49, 50]. The inhibition of MMP-9 expression in infiltrating macrophages through treatment with zoledronic acid effectively diminishes angiogenic responses in cervical cancer cells [51]. Similar to this finding, our study confirmed that TAMs that infiltrated into the tumor tissue of tumor-bearing mice are an important source of MMP-9 in the tumor environment. The decrease in MMP expression in TAMs can be an important mechanism for the inhibition of tumor angiogenesis in *Plasmodium*-infected tumor-bearing mice. The IGF/IGF-IR axis is thought to play an important role in the regulation of several MMPs and can thereby trigger MMP-mediated tumor angiogenesis and invasion [14, 15, 52, 53]. IGF-1 signals intracellularly through the PI3 and MAPK pathways after binding to IGF-1R on the cell surface, and IGF-1 thereby increases the enzymatic activity of MMP-2 and MMP-9 and enhances the proliferation and migration of tumor cells [14, 15, 54–56]. Noticeably, IGF-1 derived from polarized macrophages can maintain the M2-type activation profile in these macrophages by activating the PI3-K and MAPK pathways [54, 57]. We showed that *Plasmodium* infection significantly inhibited tumor angiogenesis by inhibiting the expression of MMP-9 in TAMs. The reduced expression of MMP-9 in TAMs was mediated by the suppression of IGF-1 via the PI3-K and MAPK pathways. These data suggest that *Plasmodium* can alter the angiogenic functions of TAMs by regulating particular signal transduction pathways.

*Plasmodium*-infected RBCs (iRBCs) were observed within the tumor vasculature in the animals. Thus, iRBCs and *Plasmodium* metabolic products, including HZ, can be easily phagocytosed by TAMs. Previous studies have suggested that the consequence of TAM phagocytosis might be the direct alteration of the activation status of TAMs, which results in decreased infiltration of TAMs into tumor sites. Alternatively, the engulfment of *Plasmodium* metabolic products induces TAM apoptosis and might thus lead to a partial depletion of TAMs. We have demonstrated that the decreased number of TAMs at least partially contributes to the inhibition of tumor angiogenesis. Additionally, our study showed that the phagocytosis of HZ by TAMs induces a significant reduction in the production of IGF-1, although the mechanism remains to be elucidated.

**Fig. 7:**
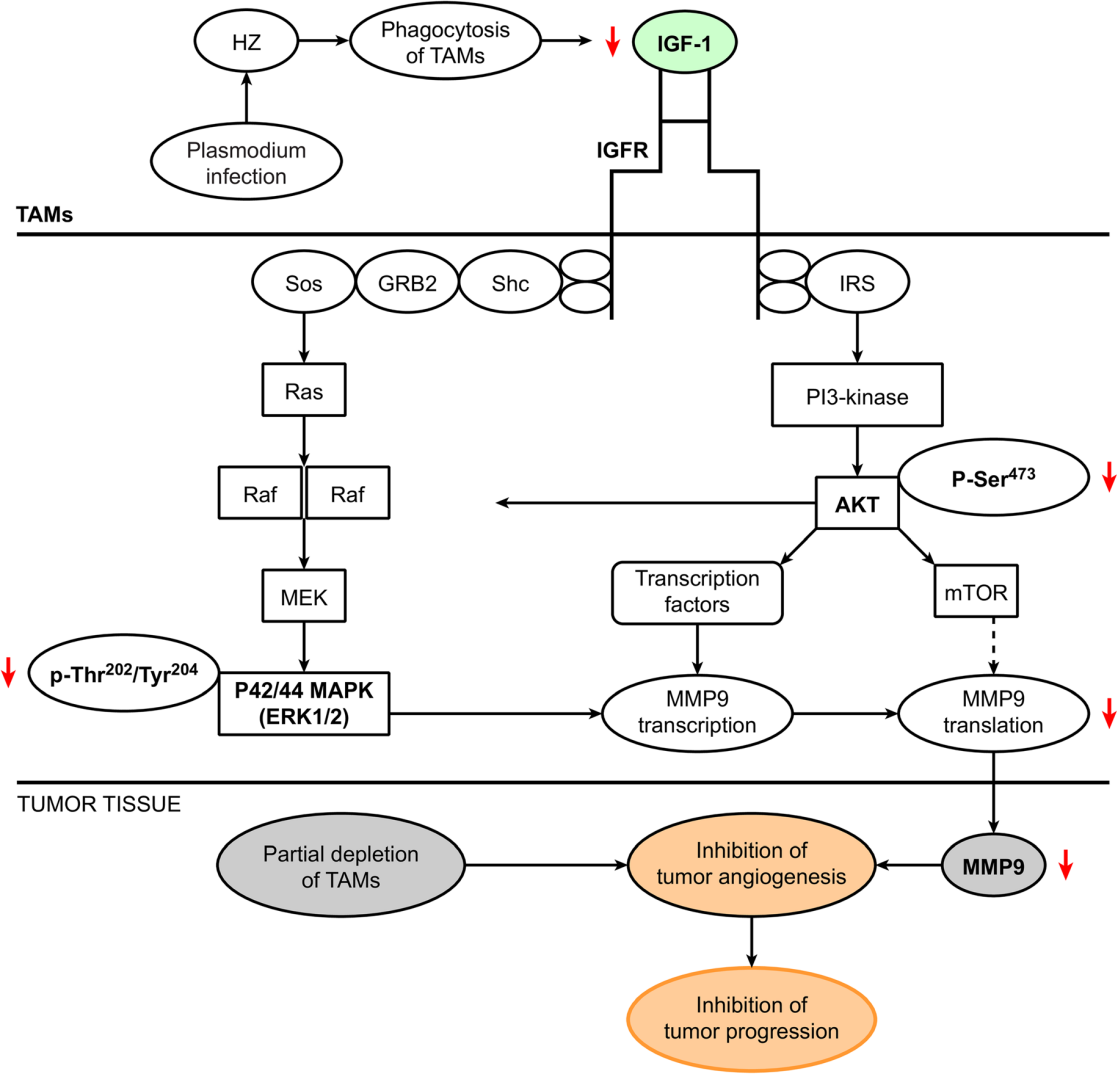
Summary of the mechanisms through which *Plasmodium* infection inhibits tumor angiogenesis by reducing the infiltration of tumor-associated macrophages (TAMs) and attenuating the expression of MMP-9 in TAMs

Overall, the present study demonstrates that *Plasmodium* infection inhibits hepatic tumor progression by reducing TAM-mediated tumor angiogenesis (Fig. 7). *Plasmodium* infection suppresses the infiltration of TAMs into tumors, which can alter the tumor milieu and thereby partially contribute to the inhibition of tumor angiogenesis. More importantly, infection can significantly reduce the levels of proangiogenic factors and increase the levels of antiangiogenic factors in TAMs. In particular, *Plasmodium* infection can significantly decrease MMP-9 expression by regulating IGF-1, which might signal intracellularly through the PI3 and MAPK pathways after binding to IGF-1R on the cell surface and thereby increase the expression of MMPs in tumor-bearing mice.

Considering the findings obtained in our previous studies [5, 8, 35, 58, 59], which showed that *Plasmodium* parasite infection induces antitumor immunity, antagonizes the tumor immunosuppressive microenvironment and inhibits tumor angiogenesis in animal models of cancer, we conclude that *Plasmodium* infection or component vaccination provides a potent therapeutic strategy for cancer treatment. Three clinical trials of *Plasmodium* immunotherapy for advanced lung cancer (clinicaltrials.gov/ct2/show/NCT02786589), advanced breast and liver cancers (clinicaltrials.gov/ct2/show/NCT03474822), and advanced cancers (clinicaltrials.gov/ct2/show/NCT03375983) have been approved and are ongoing in China. In these clinical trials, our collaborators have observed that infection with blood-stage *Plasmodium vivax* (a relatively benign form of the human malaria parasite) activates the immune system of advanced cancer patients without severe side effects or complications because artemisinin effectively controls parasitemia at a safe level (unpublished data). A gene-modified attenuated human malaria parasite could be explored as a cancer vaccine vector or an immunotherapy for cancer patients [58].

## Conclusions

In conclusion, this study highlights that the immunity mediated by *Plasmodium* infection can reactivate the immunity inhibited by tumors. Macrophages infiltrating the tumor microenvironment bridge the gap between these two different immune mechanisms. This novel method for inhibiting tumor angiogenesis through *Plasmodium* infection is of high importance for the future development of new cancer therapy strategies.

## Supplementary information

**Additional file 1: Table S1.** List of antibodies. **Table S2.** List of gene specific primers. **Figure S1.** Percentage of parasitemia after *Plasmodium* infection. **Figure S2.** Quantification of TAMs infiltrated in tumor tissue. **Figure S3.** Purities of sorted cell populations, Heatmap of the sorted TAMs, and Expression of MMP-2 and VEGF in the sorted TAMs. **Figure S4.** Immunohistochemical analysis of MMP-9 infiltrating in margin of tumor tissue by FACS-like tissuecytometer analysis system. **Figure S5.** Correlation analysis among MMP-9, TAMs, and MVD in tumor from tumor-bearing mice with or without parasite infection.

**Additional file 2.**

**Additional file 3.**

**Additional file 4.**

**Additional file 5.**

## Data Availability

The datasets generated and analyzed during this study are available from the corresponding author upon reasonable request.

## Abbreviations

TAMs: Tumor-associated macrophages
MDSCs: Myeloid-derived suppressive cells
NK: Natural killer
HCC: Hepatocellular carcinoma
LLC: Lewis lung carcinoma
HZ: Hemozoin
iRBCs: Infected red blood cells
i.p.: Intraperitoneally
s.c.: Subcutaneous
W/V: Weight versus volume
FITC: Fluorescein isothiocyanate
FBS: Fetal bovine serum
EDTA: Ethylenediaminetetraacetic acid
MAb: Monoclonal antibody
IGF-1: Type 1 insulin-like growth factor
Nppb: Natriuretic peptide B
TNF: Tumor necrosis factor
IFN: Interferon
MMP: Matrix metalloprotease
ym1: Chitinase-like 3
arg-1: Arginase 1
mgl: Macrophage galactose-type c-type lectin
fizz1: Resistin-like alpha
IL: Interleukin
inos: Inducible nitric oxide synthase
vegf: Vascular endothelial growth factor
leyve1: Lymphatic vessel endothelial hyaluronan receptor 1
Cl_2_MDP: Dichloromethylene diphosphonate
PBS: Phosphate-buffered saline
MAPK: Mitogen-activated protein kinase
PI3-K: Phosphoinositide 3-kinase
AKT: AKT serine/threonine kinase 1
p-AKT: Phospho-AKT serine/threonine kinase
PAMPs: Pathogen-associated molecular patterns
GPI: Glycosylphosphatidylinositol

## References

[1] Artavanis-Tsakonas K, Tongren JE, Riley EM (2003). The war between the malaria parasite and the immune system: immunity, immunoregulation and immunopathology. Clin Exp Immunol.

[2] Stevenson MM, Riley EM (2004). Innate immunity to malaria. Nat Rev Immunol.

[3] Greentree LB (1981). Malariotherapy and cancer. Med Hypotheses.

[4] Angsubhakorn S, Bhamarapravati N, Sahaphong S, Sathiropas P (1988). Reducing effects of rodent malaria on hepatic carcinogenesis induced by dietary aflatoxin B1. Int J Cancer.

[5] Chen L, He Z, Qin L, Li Q, Shi X, Zhao S (2011). Antitumor effect of malaria parasite infection in a murine Lewis lung cancer model through induction of innate and adaptive immunity. PLoS One.

[6] Dirkx AE, Oude Egbrink MG, Wagstaff J, Griffioen AW (2006). Monocyte/macrophage infiltration in tumors: modulators of angiogenesis. J Leukoc Biol.

[7] Klein G, Vellenga E, Fraaije MW, Kamps WA, de Bont ES (2004). The possible role of matrix metalloproteinase (MMP)-2 and MMP-9 in cancer, e.g. acute leukemia. Crit Rev Oncol Hematol.

[8] Yang Y, Liu Q, Lu J, Adah D, Yu S, Zhao S (2017). Exosomes from Plasmodium-infected hosts inhibit tumor angiogenesis in a murine Lewis lung cancer model. Oncogenesis..

[9] Lin EY, Li JF, Gnatovskiy L, Deng Y, Zhu L, Grzesik DA (2006). Macrophages regulate the angiogenic switch in a mouse model of breast cancer. Cancer Res.

[10] Wang B, Li Q, Qin L, Zhao S, Wang J, Chen X (2011). Transition of tumor-associated macrophages from MHC class II(hi) to MHC class II(low) mediates tumor progression in mice. BMC Immunol.

[11] Masson V, de la Ballina LR, Munaut C, Wielockx B, Jost M, Maillard C (2005). Contribution of host MMP-2 and MMP-9 to promote tumor vascularization and invasion of malignant keratinocytes. FASEB J.

[12] John A, Tuszynski G (2001). The role of matrix metalloproteinases in tumor angiogenesis and tumor metastasis. Pathol Oncol Res.

[13] Zhang D, Samani AA, Brodt P (2003). The role of the IGF-I receptor in the regulation of matrix metalloproteinases, tumor invasion and metastasis. Horm Metab Res.

[14] Mira E, Manes S, Lacalle RA, Marquez G, Martinez AC (1999). Insulin-like growth factor I-triggered cell migration and invasion are mediated by matrix metalloproteinase-9. Endocrinology.

[15] Saikali Z, Setya H, Singh G, Persad S (2008). Role of IGF-1/IGF-1R in regulation of invasion in DU145 prostate cancer cells. Cancer Cell Int.

[16] Chattopadhyay S, Shubayev VI (2009). MMP-9 controls Schwann cell proliferation and phenotypic remodeling via IGF-1 and ErbB receptor-mediated activation of MEK/ERK pathway. Glia..

[17] Samani AA, Brodt P (2001). The receptor for the type I insulin-like growth factor and its ligands regulate multiple cellular functions that impact on metastasis. Surg Oncol Clin N Am.

[18] Hanahan D, Weinberg RA (2000). The hallmarks of cancer. Cell..

[19] Egan TJ (2008). Haemozoin formation. Mol Biochem Parasitol.

[20] Diou J, Gauthier S, Tardif MR, Fromentin R, Lodge R, Sullivan DJ (2009). Ingestion of the malaria pigment hemozoin renders human macrophages less permissive to HIV-1 infection. Virology.

[21] Jaramillo M, Plante I, Ouellet N, Vandal K, Tessier PA, Olivier M (2004). Hemozoin-inducible proinflammatory events in *vivo*: potential role in malaria infection. J Immunol.

[22] Hoffmann J, Schirner M, Menrad A, Schneider MR (1997). A highly sensitive model for quantification of in *vivo* tumor angiogenesis induced by alginate-encapsulated tumor cells. Cancer Res.

[23] Kusmartsev S, Gabrilovich DI (2005). STAT1 signaling regulates tumor-associated macrophage-mediated T cell deletion. J Immunol.

[24] Müller-Quernheim UC, Potthast L, Müller-Quernheim J, Zissel G (2012). Tumor-cell co-culture induced alternative activation of macrophages is modulated by interferons in vitro. J Interf Cytokine Res.

[25] Pahler JC, Tazzyman S, Erez N, Chen YY, Murdoch C, Nozawa H (2008). Plasticity in tumor-promoting inflammation: impairment of macrophage recruitment evokes a compensatory neutrophil response. Neoplasia.

[26] Thomas V, Góis A, Ritts B, Burke P, Hänscheid T, McDonnell G (2012). A novel way to grow hemozoin-like crystals in vitro and its use to screen for hemozoin inhibiting antimalarial compounds. PLoS One.

[27] Scaccabarozzi D, Deroost K, Corbett Y, Lays N, Corsetto P, Salè FO (2018). Differential induction of malaria liver pathology in mice infected with Plasmodium chabaudi AS or Plasmodium berghei NK65. Malar J.

[28] Deroost K, Lays N, Pham TT, Baci D, Van den Eynde K, Komuta M (2014). Hemozoin induces hepatic inflammation in mice and is differentially associated with liver pathology depending on the Plasmodium strain. PLoS One.

[29] Kaushansky A, Ye AS, Austin LS, Mikolajczak SA, Vaughan AM, Camargo N (2013). Suppression of host p53 is critical for Plasmodium liver-stage infection. Cell Rep.

[30] Daily JP, Scanfeld D, Pochet N, Le Roch K, Plouffe D, Kamal M (2007). Distinct physiological states of Plasmodium falciparum in malaria-infected patients. Nature..

[31] Chen F, Chen J, Yang L, Liu J, Zhang X, Zhang Y (2019). Extracellular vesicle-packaged HIF-1α-stabilizing lncRNA from tumour-associated macrophages regulates aerobic glycolysis of breast cancer cells. Nat Cell Biol.

[32] Walenta S, Wetterling M, Lehrke M, Schwickert G, Sundfør K, Rofstad EK (2000). High lactate levels predict likelihood of metastases, tumor recurrence, and restricted patient survival in human cervical cancers. Cancer Res.

[33] Zhang D, Tang Z, Huang H, Zhou G, Cui C, Weng Y (2019). Metabolic regulation of gene expression by histone lactylation. Nature..

[34] Pasquetto V, Guidotti LG, Kakimi K, Tsuji M, Chisari FV (2000). Host-virus interactions during malaria infection in hepatitis B virus transgenic mice. J Exp Med.

[35] Qin L, Chen C, Chen L, Xue R, Ou-Yang M, Zhou C (2017). Worldwide malaria incidence and cancer mortality are inversely associated. Infect Agent Cancer.

[36] Liehl P, Zuzarte-Luis V, Chan J, Zillinger T, Baptista F, Carapau D (2014). Host-cell sensors for Plasmodium activate innate immunity against liver-stage infection. Nat Med.

[37] O'Neill LA, Golenbock D, Bowie AG (2013). The history of toll-like receptors - redefining innate immunity. Nat Rev Immunol.

[38] Barbalat R, Ewald SE, Mouchess ML, Barton GM (2011). Nucleic acid recognition by the innate immune system. Annu Rev Immunol.

[39] Gazzinelli RT, Kalantari P, Fitzgerald KA, Golenbock DT (2014). Innate sensing of malaria parasites. Nat Rev Immunol.

[40] van der Burg SH, Arens R, Ossendorp F, van Hall T, Melief CJ (2016). Vaccines for established cancer: overcoming the challenges posed by immune evasion. Nat Rev Cancer.

[41] Deng X, Zheng H, Zhou D, Liu Q, Ding Y, Xu W (2016). Antitumor effect of intravenous immunization with malaria genetically attenuated sporozoites through induction of innate and adaptive immunity. Int J Clin Exp Pathol.

[42] Pollard JW (2004). Tumour-educated macrophages promote tumour progression and metastasis. Nat Rev Cancer.

[43] Kuang DM, Zhao Q, Peng C, Xu J, Zhang JP, Wu C (2009). Activated monocytes in peritumoral stroma of hepatocellular carcinoma foster immune privilege and disease progression through PD-L1. J Exp Med.

[44] Kuang DM, Peng C, Zhao Q, Wu Y, Zhu LY, Wang J (2010). Tumor-activated monocytes promote expansion of IL-17-producing CD8+ T cells in hepatocellular carcinoma patients. J Immunol.

[45] Sica A, Mantovani A (2012). Macrophage plasticity and polarization: in *vivo* veritas. J Clin Invest.

[46] Chen P, Huang Y, Bong R, Ding Y, Song N, Wang X (2011). Tumor-associated macrophages promote angiogenesis and melanoma growth via adrenomedullin in a paracrine and autocrine manner. Clin Cancer Res.

[47] Sierra JR, Corso S, Caione L, Cepero V, Conrotto P, Cignetti A (2008). Tumor angiogenesis and progression are enhanced by Sema4D produced by tumor-associated macrophages. J Exp Med.

[48] Shieh YS, Hung YJ, Hsieh CB, Chen JS, Chou KC, Liu SY (2009). Tumor-associated macrophage correlated with angiogenesis and progression of mucoepidermoid carcinoma of salivary glands. Ann Surg Oncol.

[49] Lewis CE, Pollard JW (2006). Distinct role of macrophages in different tumor microenvironments. Cancer Res.

[50] Mason SD, Joyce JA (2011). Proteolytic networks in cancer. Trends Cell Biol.

[51] Giraudo E, Inoue M, Hanahan D (2004). An amino-bisphosphonate targets MMP-9-expressing macrophages and angiogenesis to impair cervical carcinogenesis. J Clin Invest.

[52] Stawowy P, Kallisch H, Kilimnik A, Margeta C, Seidah NG, Chretien M (2004). Proprotein convertases regulate insulin-like growth factor 1-induced membrane-type 1 matrix metalloproteinase in VSMCs via endoproteolytic activation of the insulin-like growth factor-1 receptor. Biochem Biophys Res Commun.

[53] Yoon A, Hurta RA (2001). Insulin like growth factor-1 selectively regulates the expression of matrix metalloproteinase-2 in malignant H-ras transformed cells. Mol Cell Biochem.

[54] Zhang M, Liu J, Li M, Zhang S, Lu Y, Liang Y (2018). Insulin-like growth factor 1/insulin-like growth factor 1 receptor signaling protects against cell apoptosis through the PI3K/AKT pathway in glioblastoma cells. Exp Ther Med.

[55] Liu L, Wang X, Li X, Wu X, Tang M (2018). Upregulation of IGF1 by tumor-associated macrophages promotes the proliferation and migration of epithelial ovarian cancer cells. Oncol Rep.

[56] Samani AA, Yakar S, LeRoith D, Brodt P (2007). The role of the IGF system in cancer growth and metastasis: overview and recent insights. Endocr Rev.

[57] Roszer T (2015). Understanding the mysterious M2 macrophage through activation markers and effector mechanisms. Mediat Inflamm.

[58] Liu Q, Yang Y, Tan X, Tao Z, Adah D, Yu S (2017). Plasmodium parasite as an effective hepatocellular carcinoma antigen glypican-3 delivery vector. Oncotarget.

[59] Adah D, Yang Y, Liu Q, Gadidasu K, Tao Z, Yu S, Dai L, Li X, Zhao S, Limei Q, Qin L, Chen X (2019). Plasmodium infection inhibits the expansion and activation of MDSCs and Tregs in the tumor microenvironment in a murine Lewis lung cancer model. Cell Commun Signal.

