# Supplementary material for: Plasmodium infection inhibits tumor angiogenesis through effects on tumor-associated macrophages in a murine implanted hepatoma model
Source: Cell Commun Signal. 2020 Sep 24;18:157. doi: 10.1186/s12964-020-00570-5 (PMC7513281; doi:10.1186/s12964-020-00570-5)
Supplement: Supplementary file 2 — Additional file 1: Table S1. List of antibodies. Table S2. List of gene specific primers. Figure S1. Percentage of parasitemia after Plasmodium infection. Figure S2. Quantification of TAMs infiltrated in tumor tissue. Figure S3. Purities of sorted cell populations, Heatmap of the sorted TAMs, and Expression of MMP-2 and VEGF in the sorted TAMs. Figure S4. Immunohistochemical analysis of MMP-9 infiltrating in margin of tumor tissue by FACS-like tissuecytometer analysis system. Figure S5. Correlation analysis among MMP-9, TAMs, and MVD in tumor from tumor-bearing mice with or without parasite infection. [file 12964_2020_570_MOESM2_ESM.pdf]

# Supplementary Material: Plasmodium infection inhibits tumor angiogenesis through effects on tumor-associated macrophages in a murine implanted hepatoma model

**Benfan Wang, Qinyan Li, Jinyan Wang, Siting Zhao, Bayaer Nashun, Li Qin, Xiaoping Chen**

**Table S1.** List of antibodies.

| Antibody | Clone | Manufacturer |
|---|---|---|
| FACS | | |
| F4/80 FITC | 11-4801 | eBioscience |
| F4/80 APC | 17-4801 | eBioscience |
| IHC | | |
| Rat anti-F4/80 | CI:A3-1 | AbD serotec |
| Rat monoclonal to Macrophage | RM0029-11H3 | Abcam |
| Rabbit anti-CD31 | ployclonal | Santa Cruza |
| Rabbit anti-MMP-9 | | CST [1] |
| WB | | |
| Mouse anti-MMP-2 | 8B4 | Santa Cruza |
| Rabbit anti-MMP-9 | ployclonal | CST [1] |
| Rabbit anti-AKT | C67E7 | |
| Rabbit anti- Phospho -AKT (Ser473) | 193H12 | |
| Rabbit anti-p44/42 MAPK (Erk1,2) | 137F5 | |
| Rabbit anti- Phospho-p44/42 MAPK (Erk1/2) (Thr202/Tyr204) | D13.14.4E | |
| Rabbit anti-VEGF | ployclonal | Abcam |
| Rabbit anti IGF-1 | | |
| HRP conjugated mouse anti-GAPDH | 6C5 | Kangcheng |

[1] CST represents Cell Signaling Technology

**Table S2.** List of gene specific primers

| Gene | Forward primer | Reverse primer | Ref. |
|---|---|---|---|
| *Ym1* | GGGCATACCTTTATCCTGAG | CCACTGAAGTCATCCATGTC | [1] |
| *Fizzl* | TCCCAGTGAATACTGATGAGA | CCACTCTGGATCTCCCAAGA | |
| *Arg 1* | ATGGAAGAGACCTTCAGCTAC | GCTGTCTTCCCAAGAGTTGGG | [2] |
| *Mgl 1* | AACCTCCAGAACTCAAGGATCG | AGCTTTACCAGGCTCTTGGGT | [3] |
| *Mgl 2* | CAGAACTTGGAGCGGGAAGAG | TTCTTGTCACCATTTCTCATCTCCT | |
| *iNOS* | GCTTCTGGTCGATGTCATGAG | TCCACCAGGAGATGTTGAAC | [4] |

| | | |
|---|---|---|
| *IL-12B* | GAAAGACCCTGACCATCACT | CCTTCTCTGCAGACAGAGAC |
| *Leyvel* | CTGGCTGTTTGCTACGTGAA | CATGAAACTTGCCTCGTGTG |
| *VEGFA* | CAGGCTGCTGTAACGATGAA | AATGCTTTCTCCGCTCTGAA |
| *MMP-2* | GAATGCCATCCCTGATAACCT | GCTTCCAAACTTCACGCTCTT |
| *MMP-9* | GGCAACGGAGAAGGCAAAC | CCACTCGGGTAGGGCAGAA |
| *IGF-1* | CGCTCTGCTTGCTCACCTT | TCATCCACAATGCCTGTCT |
| *β-Actin* | GGCTGTATTCCCCTCCATCG | CCAGTTGGTAACAATGCCATGT |

## Reference


1. Raes G, De Baetselier P, Noel W, Beschin A, Brombacher F, Hassanzadeh G. Differential expression of FIZZ1 and Ym1 in alternatively versus classically activated macrophages. J Leukoc Biol. 2002,71:597–602.
2. Raes G, Brys L., Dahal BK, Brandt J, Grooten J, Brombacher F, Vanham G, Noël W, Bogaert P, Boonefaes T, et al. Macrophage galactosetype C-type lectins as novel markers for alternatively activated macrophages elicited by parasitic infections and allergic airway inflammation. J Leukoc Biol. 2005,77:321-27.
3. Singh SK, Streng-Ouwehand I, Litjens M, Weelij DR, García-Vallejo JJ, van Vliet SJ, Saeland E, van Kooyk Y. Characterization of murine MGL1 and MGL2 C-type lectins: distinct glycan specificities and tumor binding properties. Mol Immunol. 2009,46:1249.
4. Movahedi K, Laoui D, Gysemans C, Baeten M, Stangé G, Van den Bossche J, Mack M, Pipeleers D, In't Veld P, De Baetselier P, et al. Different tumor microenvironments contain functionally distinct subsets of macrophages derived from Ly6C(high) monocytes. Cancer Res. 2010,70:5728-39.


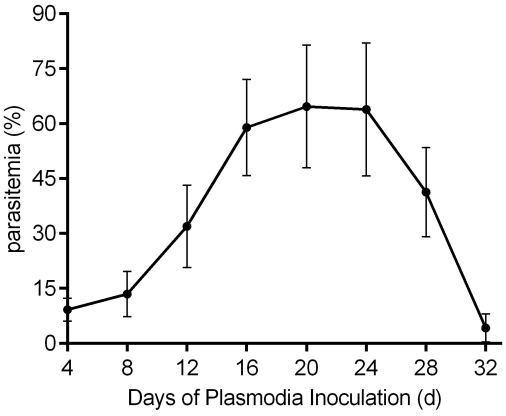


**Figure S1.** Percentage of parasitemia after *Plasmodium*. The percentage (parasite infected red blood cells per 100 red blood cells) of parasitemia in tumor-bearing mice after *Plasmodium* infection was monitored during the progression of the *Plasmodium* infection by Giemsa staining of blood smears obtained on an every-four-day basis.

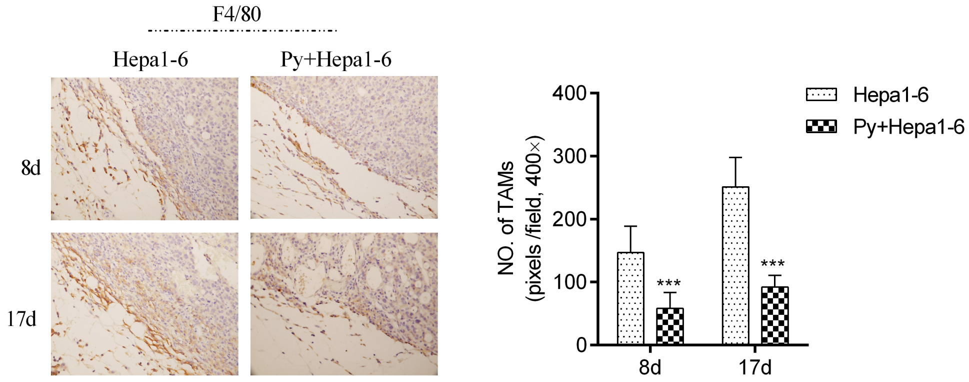


**Figure S 2.** Quantification of TAMs infiltrated in tumor tissue. Tumor histological specimens were prepared on days 8 and 17 after infection of the *Plasmodium* parasites (n=4) and stained immunohistochemically with F4/80 mAb (colored by DAB). The representative images were presented. The number of infiltrating TAMs was quantified by counting. ***, $p < 0.001$.

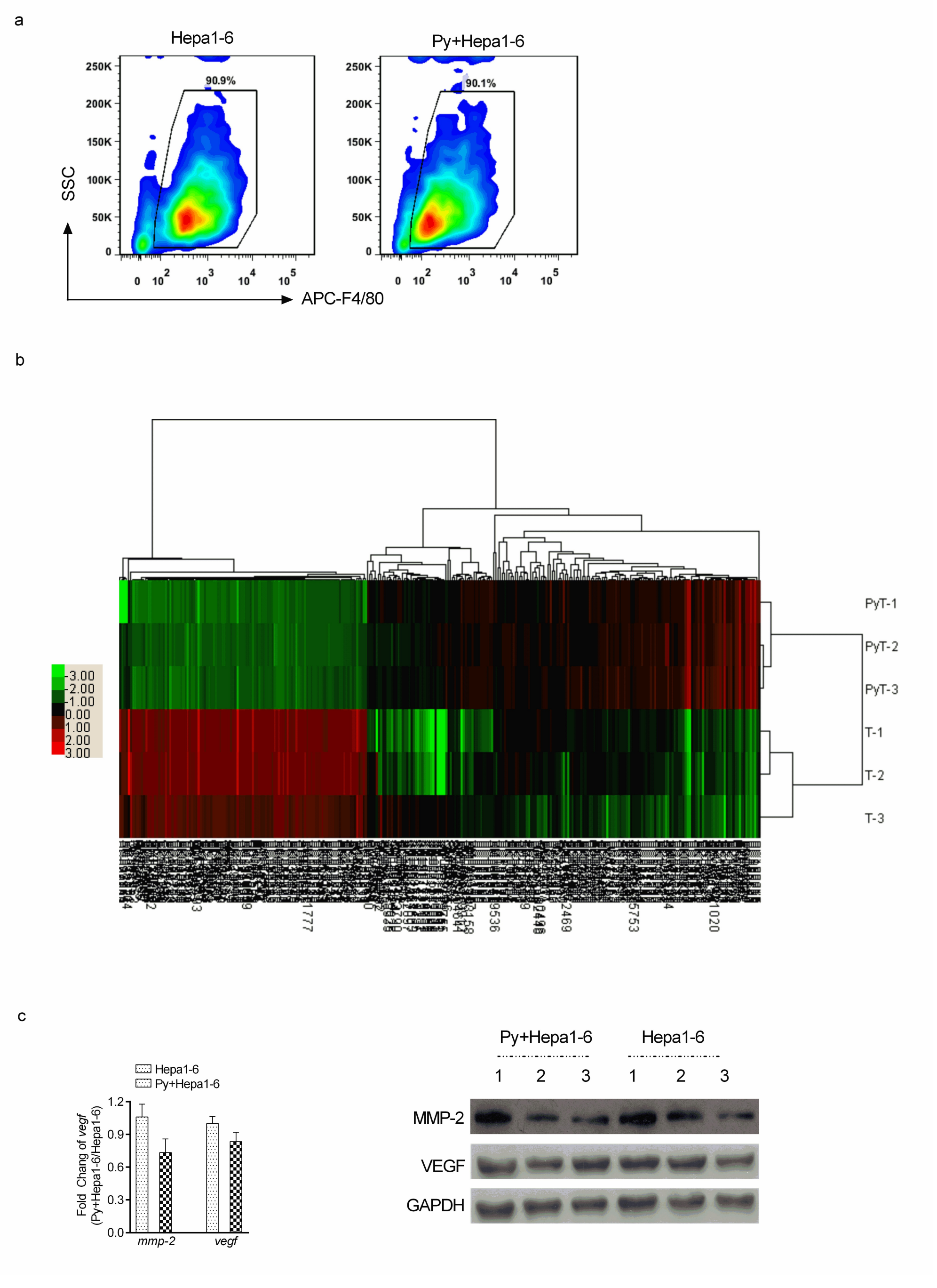


**Figure S3.** Purities of sorted cell populations, Heatmap of the sorted TAMs, and Expression of VEGF in the sorted TAMs. (a) The FACS-sorted cell populations that were used throughout the study are shown in the representative smooth plots. F4/80$^{+}$ TAMs were purified from the hepa1-6 cell-implanted tumor-bearing mice infected or uninfected with *Plasmodium*. (b) Heat map of the sorted TAMs form tumor-bearing mice on day 17 after *Plasmodium* infection compared to the sorted TAMs from uninfected tumor-bearing mice. (c) The tumor-bearing mice were sacrificed on day 17 after *Plasmodium* infection (n=3). Total RNA and protein were extracted from the sorted TAMs. Relative quantification of *mmp-2* and *vegf* was performed using qRT-PCR (left panel). The level of MMP-2 and VEGF protein was assessed by immunoblot analysis (right panel).

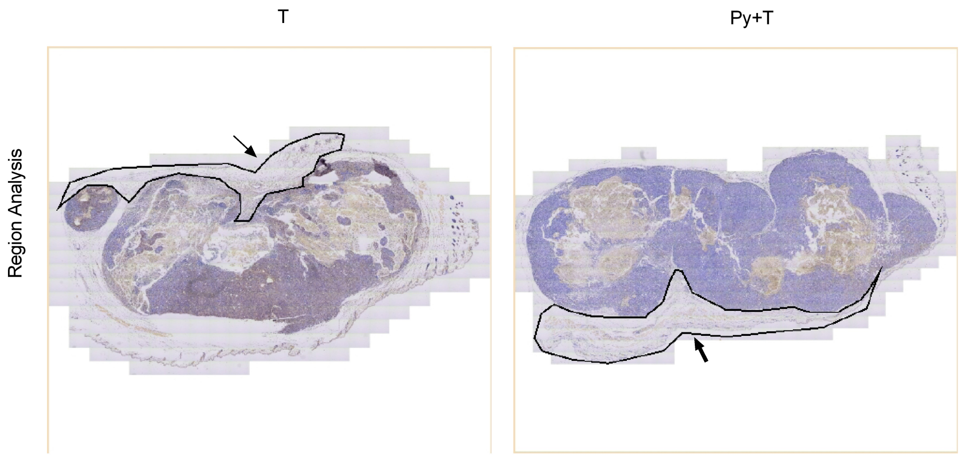


**Figure S4.** Immunohistochemical analysis of MMP-9 infiltrating in margin of tumor tissue by FACS-like tissuecytometer analysis system. A majority of TAMs infiltrated in the margins of tumor tissues in the s.c. transplanted tumor-bearing mice. The expression of MMP-9 was assessed by immunohistochemistry (n=4). A representative section was analyzed using FACS-like tissuecytometer analysis system. The typical expression area of the margin was chosen for analysis (×200 magnification). The size of the area was 8.672664 mm2 for tumor sections from tumor-bearing mice with *Plasmodium* infection and 3.877938 mm2 for tumor sections from tumor-bearing mice without infection.

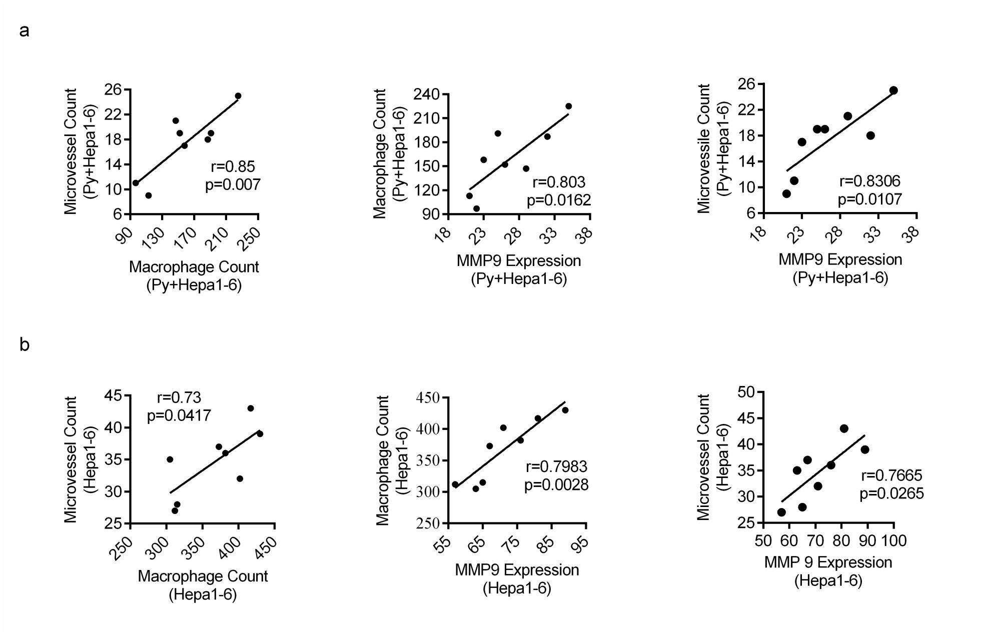


**Figure S5.** Correlation analysis among MMP-9, TAMs, and MVD in tumor from tumor-bearing mice with or without parasite infection. (a-b) Correlation analysis among MMP-9, TAMs, and MVD in tumor tissues from tumor-bearing mice on day 17 after *Plasmodium* infection (a) and uninfected tumor-bearing mice was carried out using Pearson's correlation (b).
